# Association of the Length of Service of 24/48 Firefighters with the Quality of Their Diet and Selected Anthropometric Parameters

**DOI:** 10.3390/nu15184029

**Published:** 2023-09-17

**Authors:** Karolina Dobrowolska-Zrałka, Krzysztof Kujawa, Bożena Regulska-Ilow

**Affiliations:** 1 Department of Dietetics and Bromatology, Wroclaw Medical University, ul. Borowska 211, 50-556 Wroclaw, Poland; bozena.regulska-ilow@umw.edu.pl; 2 Statistical Analysis Centre, Wroclaw Medical University, ul. K. Marcinkowskiego 2-6, 50-368 Wroclaw, Poland; krzysztof.kujawa@umw.edu.pl

**Keywords:** firefighters, shift work, Nutrient-Rich Food Index, diet quality, body composition

## Abstract

The aim of the study was to examine the association of lengths of service (LS) ≤ 10 years and >10 years in 24/48 shifts with the quality of the observed diet based on the Nutrient Rich Food Index (NRF9.3) and selected anthropometric parameters of 130 firefighters of the State Fire Service (SFS) in Wroclaw, Poland. The study also analysed the individual components of the men’s diets required to calculate the NRF9.3 index in both seniority groups. Men with LS > 10 years had statistically significant higher body weight (89.00 kg vs. 81.59 kg), body-fat level (22.80 kg vs. 17.95 kg), waist circumference (96.50 cm vs. 89.00 cm), body-fat percentage (21.94 ± 4.06% vs. 25.00 ± 5.45%), body mass index (BMI) (28.10 kg/m^2^ vs. 25.40 kg/m^2^) and waist–hip ratio (WHR) (0.84 0.92 vs. 0.84), compared to the LF ≤ 10 years group. In contrast, the quality of the men’s dietary adherence, based on the calculated NRF9.3 index, did not differ between the study groups, and was 662.50 ± 103.1 and 664.78 for the LS ≤ 10 years and LS > 10 years groups, respectively. Based on a statistical analysis using the NRF9.3 diet quality index by tertile (NRF9.3-C), a leading and statistically significant association of LS > 10 years vs. ≤10 years was observed as to most of the anthropometric parameters studied. In contrast, the association of participants’ diet quality, as assessed by the NRF9.3 index value, was insignificant for all anthropometric parameters studied.

## 1. Introduction

The definition of night shift work refers to work at times other than the most common daytime hours (i.e., approximately 9 a.m. to 5 p.m.), and is defined differently, depending on the sources [1,2]. However, shift work refers to 24 h shifts, evening or night shifts, rotating shifts, and on-call shifts. Irregular schedules (both day and night work) are also included in the definition [2].

The prevalence of night-shift work varies according to occupational industry; however, night work is most common in health care, production, transport, retail, and service industries. According to the International Agency for Research on Cancer (IARC), disruption of the circadian rhythm in night shift workers, including intercontinental travellers, is the result of disruption of the cycle of day and night, mainly the light–dark cycle [3].

According to Seo M. et al. [4], the firefighting profession has a high risk of injury and even fatal accidents, so comprehensive elimination of risk factors is vital for the safety and health of firefighters [4]. The authors of previous scientific studies claim the tasks firefighters have to perform in the course of their professional duties require, for example, appropriate body composition (e.g., low levels of body fat) and muscular strength and endurance [5]. According to the definition, metabolic syndrome is the presence of abdominal obesity and a minimum of two of the following criteria: elevated blood pressure, abnormal glucose metabolism, and atherogenic dyslipidaemia [6]; this list has been defined as a group of risk factors for cardiometabolic diseases [7].

The diagnosis of metabolic syndrome among firefighters may indicate a predisposition to the occurrence of, for example, cognitive impairment, even when a normal BMI is found; thus, the prevention or treatment of metabolic syndrome may increase the safety and efficiency of firefighters [4]. Effective lifestyle changes are crucial in the prevention and treatment of metabolic syndrome, including the primary risk factor for the development of the disorder, i.e., overweight status or obesity, as well as associated disease entities. Of particular therapeutic importance are healthy eating and the elimination of the use of stimulants [6].

Most scientific studies published in this area to date involving firefighters concern the importance of occupational stress and burnout [8,9], the prevalence of cardiovascular disease in this occupational group [4,10,11,12,13,14] and obesity [15,16]. In Poland, scientific research efforts with the participation of State Fire Service firefighters are also limited and mainly refer to the analysis of sleep disorders [17], post-traumatic stress and burnout [18], or the frequency of accidents during work [19,20]. Based on our current knowledge, no scientific studies analysing the association of shift work with diet quality and selected anthropometric measurements of firefighters have been conducted. The scientific studies we analysed on the diet of firefighters were related to nutritional interventions to reduce the participants’ blood pressure [12] or excessive body weight [21], or to check the factors influencing food intake during the day and night shifts, together with an assessment of the types of food and beverages consumed and the timing of meals on working days [22]. An analysis of dietary intake was carried out by Torre S. et al. [23]; however, this only concerned kilocalories, macronutrients (protein, fat, saturated fatty acids and carbohydrates), added sugars, dietary fibre and alcohol. The study additionally examined the men’s adherence to proper nutrition based on the Plan National Nutrition et Santé Guideline Score, but only for a limited number of participants (*n* = 28) [23].

The aim of this study was to analyse the association of selected anthropometric measurements and diet quality based on the calculated NRF9.3 index with the length of service being ≤10 years vs. >10 years for 130 firefighters of the State Fire Service, who were employed in firefighting stations in Wrocław, Poland, working in 24/48 shifts. The individual components of the men’s diet required to calculate the NRF9.3 index in both groups were also analysed.

## 2. Materials and Methods

### 2.1. Participants

All men employed in the eight Fire and Rescue Units of the State Fire Service in the city of Wrocław, Poland, representing a group of approximately 383 men, were invited to participate in our study. Invitations were delivered as information leaflets distributed at firefighter meetings at each fire station, following approval from the Commander. The men work a 24/48 shift system (24 h on duty, 48 h off) and additionally, once per month, they have what is known as ‘free duty’, meaning a day off when the other colleagues from the shift are in the firefighting station.

The number of participants in the study was 133, with men from 7 units, representing approximately 34.73% of all firefighters employed by SFS in the city of Wrocław, and 130 of them (97.74% of the men enrolled and approximately 33.94% of all firefighters in SFS in the city of Wrocław) prepared a 3-day food diary. A detailed description of the dietary intake of the men is provided in the next section (Data Collection and Research Methods Used).

The men were divided into two groups, in terms of length-of-service (LS). The group with short seniority comprised participants performing their job ≤ 10 years (*n* = 63 by anthropometric analysis and *n* = 61 by diet analysis of participants), and the group with long seniority comprised participants performing their job > 10 years (*n* = 71 by anthropometric analysis and *n* = 69 by diet analysis of participants).

### 2.2. Data Collection and Research Methods Used

The study was conducted in accordance with the Declaration of Helsinki and approved by the Institutional Ethics Committee of Wroclaw Medical University, (Nr KB - 760/2020).

From November 2021 to April 2022, after receiving permission from the City Commander of the State Fire Service in Wrocław and the individual Fire and Rescue Unit Commanders, as well as determining the willingness of the firefighters to participate in the study, a convenient date was set for measurements to be taken on the premises of the respective Fire and Rescue Units for all three shifts. On the day of the study, each candidate, after being briefed on the details of the study (its purpose, conduct and possible risks and benefits), how the Medical University collects personal data, and being covered by the University’s research activities insurance, signed a form indicating their consent to participate voluntarily in the study.

In the next step, the men were measured for body composition analysis (body weight, muscle and fat tissue; BMI and waist–hip Ratio (WHR); total body water content, intracellular water content and extracellular water content; extracellular-to-total-body water ratio; and impedance) using a portable body composition analyser, Accuniq BC310 (Selvas Healthcare, Sinseong-ro, Yuseong-gu, Daejeon, 34109, Republic of Korea); for height, with a TANITA HR-001 mobile stadiometer (TANITA, Tokyo, Japan); for waist circumference using a GIMA BMI measuring tape; for blood pressure with an Omron Healthcare Co. blood pressure monitor, M6 Comfort, HEM-7360-E (Kyoto, Japan); and for muscle strength with a kern mat dynamometer, version 1.2 (KERN & SOHN GmbH, Balingen, Germany).

A dietary intake interview was conducted with each study participant, based on prepared food diaries, and taking into account the foods eaten and drinks consumed between awakening and falling asleep. Dietary intake included 2 days on duty and 1 day off. In order to determine the portion sizes of individual products and meals, the study volunteers used household measures (e.g., spoon, glass and bowl); additionally, during the dietary interview, the Photo Album of Products and Foods was used to detail serving sizes [24].

Based on the participants’ collected dietary interviews, an analysis was conducted using tables of food composition [25] and nutritional value and the computer programme “Food Processor ESHA Research” (USA). Following the data obtained from the analysis, the next stage of the study assessed the quality of the firefighters’ diets, using the Nutrient Rich Food Index (NRF), taking into account 9 nutrients to be encouraged (protein, dietary fibre, vitamins A, C and D and minerals, specifically, calcium, iron, potassium and magnesium) and 3 nutrients to limit (saturated fatty acids, added sugar and sodium) (NRF9.3) [26,27].

### 2.3. Nutrient Rich Food Factor Calculation (NRF9.3)

The nutrient-rich food factor is used to estimate the nutrient density of a diet and is used as a key component of dietary advice. The NRF can be used to assess the nutritional value of an individual food, a single meal, or an overall diet [26].

The variant NRF9.3, which was used in the study, is based on the difference in the sums of the percentages of daily values for the nine components desired in the diet and the three components whose intake should be limited [26].

The total metabolism (TM) of each firefighter was calculated individually, based on basal metabolism obtained from body composition analysis using a body composition analyser and multiplied by a physical activity level (PAL) index of 1.75, appropriate for those with a job involving a higher energy expenditure than sedentary work and performing regular moderate-to-vigorous physical activity [28]. In addition to their firefighting duties, SFS officers perform tactical drills while on duty and are involved in grocery shopping, meal preparation and housekeeping work on the station (e.g., repairs, keeping the station and rescue equipment clean and minor repair work on firefighting vehicles). Moreover, almost all of the men declared having a second job, mostly physical in nature.

A protein energy intake was calculated based on the recommendations of the Institute of Food and Nutrition (IFN) [29]. According to the IFN [29], the protein energy percentage in the total daily ration in the diet of men should be between 10–15% of the total energy requirements. For NRF9.3 calculations, a 10% proportion of protein in firefighters’ diets was taken as the minimum, proper energy proportion of this macronutrient in the diets of adult men [29]. Saturated fatty acids (SFA) and added sugars (AS), however, were determined based on WHO guidelines recommending that their intake should account for <10% of total energy intake [30,31].

Next, the requirements of their dietary needs were calculated using the Recommended Dietary Allowances (RDA) developed by the IFN [29] based on the recommendations of the World Health Organisation (WHO) [31] and the European Food and Safety Authority (EFSA) [32] (Table 1). For this purpose, an NRF9.3 index was calculated, and the percentage meeting the RDA was capped at 100 for desirable dietary components so that a high intake of one component did not compensate for a low supply of another. Conversely, if a calculated value for an ingredient whose contribution to the diet should be limited had a negative value (when its dietary intake was less than the maximum allowed), then a value of 0 was taken for that ingredient. In analysing the proportion of dietary components whose intake should be restricted, the percentage exceeding the limit values was used to calculate the index [33,34].

The NRF9.3 index for each participant’s diet was calculated according to the following formula, where:

- sgn—sign function, returns a value of −1 for negative numbers, a value of 0 for the number 0 and a value 1 for positive numbers;
- mod—modulo function, returns the remainder of dividing the number preceding the ‘mod’ expression by the following number;
- div—divide function returns the integer from dividing the number preceding the ‘div’ expression by the following number;
- x_i_—means the amount of each ingredient encouraged in the diet;
- y_m_—means the amount of an ingredient whose intake should be limited;
- RDA_x_—RDA for ingredient x, as encouraged in the diet;
- MRVy—is the MRV for ingredient y, the contribution of which in the diet should be limited.

$${NR}_{9}=\sum_{i=1}^{9}100\left\{sgn\left[sgn\left(\frac{x_{i}}{{RDA}_{x}}-1\right)+1\right]-\frac{x_{i}\;mod{\;RDA}_{x}}{{RDA}_{x}}\;\cdot sgn\left[sgn\left(x_{i}\;div\;{RDA}_{x}\right)-1\right]\right\}\;{NR}_{3}=\sum_{m=1}^{3}100\cdot y_{m}\;div\;{MRV}_{y}+100\cdot sgn\left[sgn\;\left(\frac{y_{m}}{{MRV}_{y}}-1\right)\right]\cdot \left(\frac{y_{m}\;mod{\;MRV}_{y}}{{MRV}_{y}}-1\right)$$

The above formulas can also be written as formulas in Excel, as follows. 

For NR9:

= 100 * (SIGN(SIGN((x_i_/RDA_i_ − 1) + 1) − MOD(x_i_;RDA_i_)/RDA_i_ * SIGN(SIGN(QUOTIENT(x_i_;RDA_i_)) − 1))

For NR3

= 100 * QUOTIENT(y_m_;MRV_y_) + 100 * SIGN(SIGN(y_m_/MRV_y_ − 1) + 1) * (MOD(y_m_;MRV_y_)/MRV_y_ − 1)

The higher the value of calculated NRF9.3, the higher the quality of the analysed diet. The maximum score is 900, which suggests that, for a given energy supply, the RDA has been met or exceeded for nine desirable dietary components, while the recommended restriction of undesirable dietary components has been achieved [33,34,35].

For a simplified calculation of the NRF9.3 indicator, please refer to Supplementary Table S1.

## 3. Statistical Analysis

The conformity of the distribution of values in a given group to a normal distribution was assessed using the Shapiro–Wilk test. If the distribution was statistically significantly different from the norm, the non-parametric Mann–Whitney U test was used in the analysis of differences between groups; in the case of a normal distribution, the t test was used.

The diet quality index NRF9.3 was used in the statistical analysis by tertile, as shown in Table 2.

The association of anthropometric characteristics with LS and the tertile of the diet quality index (NRF9.3-C) was analysed using two-factor analysis of variance with interaction, which was included to recognize whether diet quality and length-of-service affected an explained variable independently. Depending on whether the assumption of normal distribution was or was not met, the analysis was performed using parametric analysis of variance (ANOVA) and non-parametric robust ANOVA, respectively. For ANOVA, homogeneity of variances was checked using Levene’s test, and in almost all cases the assumption was met, except for Imp., in which the variances between centile groups differed (F_2, 127_ = 4.61, *p* = 0.012).

Non-parametric analysis was performed with the R package “WRS2” [36] using the command t2way(formula = “*parameter*” ~ LScode + NRFtertiles + LScode:NRFtertiles, data = dane). This resulted in the determination of its p-values, indicating the statistical significance (or lack thereof) of the association between a given explanatory variable (the “parameter”) and length of tenure (Fscode), diet quality (NRFtertiles), or the interaction between the two.

Statistical analysis was performed using Statistica PL 13.3 from StatSoft (StatSoft Inc., Tulsa, OK, USA) and the WRS2 package (Mair and Wilcox 2020) in the R 4.1.2 environment.

## 4. Results

Significant anthropometric differences were observed between the groups with short and long seniority, as shown in Table 3. Men in the LS > 10 years group had statistically significant higher body weight, body fat, waist circumference, body fat percentage, BMI and WHR relative to the LS ≤ 10 years group.

A lack of statistically significant differences between the groups was only noted as to height, muscle strength (for the right and left arms) and the participants’ body-water content (total, extracellular and intracellular).

A comparison of the study participants’ diets, including the average NRF9.3, by short and long service, is shown in Table 4. Firefighters’ diets from the LS ≤ 10 years and LS > 10 years groups differed with statistical significance in the percentage of energy from protein (18.86 ± 3.4 vs. 17.45 ± 2.9), ratio of omega-6 to omega-3 fatty acids (5.63 (3. 93, 7.11) vs. 4.18 (3.41, 6.10)), vitamin B6 (3.04 (2.46, 3.49) vs. 2.59 (2.06, 3.50)), vitamin C (144.76 (94.36, 196.62) vs. 107.350 (71.86, 149.14)) and folate (442.20 (358.06, 574.67) vs. 363.82 (290.89, 475.90)). Other participants’ diet-related variables were not statistically significantly different between groups.

In this study, the individual dietary components required to calculate the diet quality index NRF9.3 were also analysed. No statistically significant differences between the groups of men LS ≤ 10 years and LS > 10 years (according to the Mann–Whitney test) in the assessment of diet quality and individual dietary components were observed. These results are presented in Table 5.

In the LS ≤ 10 years group, men’s diets received 662.50 ± 103.1 points, with LS > 10 years receiving 664.78 (602.67, 710.41), but from a separate analysis of the calculated diet quality index NRF9.3, it was observed that more than 800 points were received by the diets of six men (4.62%), four for LS ≤ 10 years and two for LS > 10 years. However, NRF9.3 scores between 700–800 points were received by the diets of thirty-eight men (29.23%) (twenty with ST ≤ 10 years, and eighteen with LS > 10 years). Below 500 points, only thirteen men (10.00%) received such diets (five for ST ≤ 10 years, and eight for LS > 10 years).

None of the men’s diets exceeded the allowable maximum for added sugars in the diet. In addition, all LS men ≤ 10 years exceeded the sodium supply with their diets. The percentage of participants, compared between the short- and long-term length-of-service groups, whose diets provided the requirements for the individual components desirable in the diet and the dietary recommendations to limit the components not desirable in the diet, together with the calculated Chi2 test, are shown in Table 6. Only for SFA were the differences between men in the seniority groups ≤ 10 years and > 10 years statistically significant, with a higher proportion in the seniority group ≤ 10 years.

Based on a statistical analysis using the NRF9.3 diet quality index by tertile (NRF9.3-C), a leading and statistically significant effect of length-of-service was observed as to most of the parameters studied. In contrast, the significance of the calculated diet quality of the study participants was negligible (*p* > 0.05) for all parameters tested. Only for impedance was the result of the statistical analysis for NRF9.3 borderline significant (*p* < 0.1), and it decreases in successive tertiles, as shown in Table 7 and Table 8.

A significant association was observed between length-of-service (≤10 years vs. >10 years) and the anthropometric measures studied, such as BW, WC, BMI, WHR, SMM, FM, TBW, ECW, and ECW ind. There was no statistically significant association between seniority and anthropometric characteristics such as RA, ICW and RCT.

The quality of the diet described by the NRF9.3-C index (with a breakdown into tertiles) was not related to either anthropometric characteristics or seniority. Similarly, there were no differences between NRF9.3-C index values between the different tertiles (Low, Medium and High).

## 5. Discussion

Our results show a strong correlation between shift-work seniority and anthropometric measurements of National Fire Service firefighters in Wrocław, with no statistically significant differences in diet quality between men with LS ≤ 10 years and LS > 10 years. Anthropometric parameters—especially waist circumference, WHR, BMI and FM, and PBF—are key risk factors for metabolic syndrome (MetS). The significance of shift work on the increased risk of this disorder was also confirmed in a meta-analysis by Wang Y. et al. [37] and Khosravipour M. et al. [1]. In addition, Khosravipour M. et al. [1] noted that groups of subjects performing their work duties on a rotating basis, i.e., both day and night hours, had a higher risk of MetS, compared to those working in other shift patterns. The authors consider the permanent modification of working hours, longer shifts and higher incidence of night shifts among rotating shift workers to be responsible for the higher risk of MetS diagnosis [1]. According to the cited MetS definition, maintaining a proper body weight and waist circumference are fundamental in preventing the development of metabolic syndrome, and this can be achieved with an age-, height- and activity-appropriate nutritious and balanced diet.

The anthropometric results we have presented are consistent with those published by Choi B. et al. [15]. BMI was statistically significantly higher in older firefighters and thus those with longer seniority. In addition, the authors of the study observed that the more often the study participants were called out on emergency calls per month during their service, the more frequently these men were reported to be obese (based on BMI, WHR or percentage-body-fat based on skinfold measurements) [15]. Comparing our results with the observations of Choi B. et al. [15], we might suspect that the number of actions in which men are involved, which is closely related to seniority, may be important in the prevalence of obesity among professional firefighters.

The data we collected showed that men with > 10 years of service were characterised by statistically significant higher BMI, WHR, waist circumference and body fat (both expressed in kilograms and percentage), compared to men with shorter lengths of service in the SFS. Anthropometric measurement analysis of men with LS > 10 years indicated a significantly higher prevalence of overweight status or obesity in this group of men, which is a risk factor for, among other things, cardiovascular disease and MetS. The increased-body-weight phenomenon in firefighters has also been observed in studies in the United States [38], France [39], Portugal [16] and Brazil [40].

The finding of overweight status and/or obesity in firefighters regularly exercising would be a mistake if it referred solely to the BMI of the study participants, due to the latter’s association with both fat and lean body mass [41]. Obesity, as determined by BMI alone, is also diagnosed in individuals with lower body fat and a higher proportion of muscle tissue [42]. Therefore, in our study, when analysing the anthropometric differences between the seniority groups, we also examined the participants’ WC, WHR, PBF and FM. All of these parameters were statistically significantly higher in the seniority group of >10 years, compared to the seniority group ≤ 10 years.

The importance of length-of-service on firefighter health has also been shown by Wolffe T.A.M. et al. [43]. The authors reported that the cancer rate among men aged 35–39 years was 323% higher than in the general population. In addition, the risk was higher in those with longer work experience ≥ 15 years, compared to men working for less time [43].

Souza R.V. et al. [44], in their meta-analysis, concluded that excessive intake of saturated fatty acids and soft drinks, which was often observed in shift workers, were recognised as metabolic risk factors contributing to the development of overweight status, obesity and non-genital chronic diseases. Additionally, changes in meal regularity and breakfast skipping were among the independent factors for weight gain and obesity, even despite a proper kilocalorie supply within the diets among the participants [44].

The National Health and Nutrition Examination Survey published analyses of data showing that diets with higher NRF scores were characterised by a higher proportion of desirable foods in the diet, higher Healthy Eating Index (HEI) values and lower energy intake. Fulgoni V. et al. [27] observed in their study that diet quality assessments performed using the NRF index were significantly associated with HEI scores, and a variation of NRF9.3 may serve to further develop this algorithm [27]. Additionally, in a cross-sectional study involving Dutch adults, the authors found no significant differences in diet-quality scores using the NRF index, compared to the Dutch Healthy Diet Index (DHD). The researchers also highlighted that, of the fifteen types of NRF indices analysed, the NRF9.3 version, per 100 kcal, showed the greatest prediction of DHD index with the R^2^ model [45]. Additionally, van Lee L. [46] noted in her study that higher DHD scores were significantly associated with a lower risk of mortality among participants [46]. Furthermore, from the analysis of data collected in the Rotterdam Study, it was observed that NRF9.3 values were inversely correlated with the incidence of cardiovascular disease, but these results were not statistically significant [47].

Cited studies confirm the validity of using the NRF9.3 index to assess the quality of participants’ diets. However, due to the differences in age, height and weight among the study’s participants, consistent standards could not be used, as was the case when calculating the quality of a single food product for food labelling purposes. In that case, the NRF9.3 index referred to the nutritional assumptions developed for a diet with a 2000 kcal energy value [34,35,47].

The protein requirements of each study participant and the maximum allowable values for dietary AS and SFA were calculated individually based on the TM. The participants’ TM was 2338.00–3575.25 kcal/day, with 10% of energy being from protein and accounting for 58.45–89.38 g/day, respectively. Similarly, we proceeded with the calculation of the maximum dietary contribution of AS and SFA; the guidelines require AS and SFA to account for <10% of the TM, which was <58.45–89.38 g and <25.98–39.73 g, respectively.

Protein requirements relate to body weight or percentage of energy; therefore, we recognised that it would be a mistake to have the same protein standard, expressed in grams, for all 130 firefighters. Considering the type of work performed, the assumption, as per food labelling, of a sufficient protein intake of 50 g, in our opinion would be incorrect and insufficient for the participants in our study. The allowable values, expressed in grams, may even be nearly twice as high for some men as for a person whose energy requirement is 2000 kcal.

Based on the scores obtained from the individual components of the index, as well as separately for the calculated total nutrients encouraged and limited in the diet, it is possible to represent an overview of the dietary habits of an individual and, as in our study, of a group of men divided by length of service. The Wrocław firefighters’ diet is low in dietary fibre, which may suggest an intake of highly processed cereal products (e.g., wheat bread and pasta) and a low vegetable intake (especially for raw vegetables). On the other hand, the high percentage of participants whose diets fulfilled the iron requirements and exceeded the maximum allowable sodium intake might be expected to have a high consumption of, for example, processed meat products (e.g., sausages, pâtés and cold cuts).

However, the assessment of this professional group’s diet based on the NRF9.3 index has some limitations. The main and crucial factor that can interfere with a genuine assessment of the quality of the diet is represented by the aforementioned high energy requirements of the study group. The men’s total metabolism, due to their job duties and anthropometric build, averaged over 2800 kcal for both seniority groups. High food intake is associated with a lower risk of nutritional deficiencies, relative to a lower energy diet. As a result, the NRF9.3 indicator assesses the extent to which the nutrient requirements are fulfilled, rather than the quality of the diet, in the sense of including nutrient-dense foods. As stated by Fulgoni V. et al. [27], high-scoring diets as to calculated diet quality indicators are not necessarily due to a reduction in certain nutrients whose supply should be limited. The authors state that the addition of the encouragement of certain nutrients in the diet is fundamental to achieving higher scores in the calculation of the diet quality index [27].

In addition, as to the encouragement components of the indicators in the diet, their permissible intake is not included. The NRF9.3 dietary assessment is focused on fulfilling the minimum requirement for vitamins, minerals and protein, but does not take into account their over-supply—e.g., too much protein intake at a time of the increasing popularity of building muscle mass and increasing diagnosis of kidney disease—which would be very valuable information [48]. Analogous to this are the dietary components to be limited, mainly sodium [49,50].

The results obtained in our study are different, relative to previously published data. Fritschi et al. [51] and Wang et al. [52] claimed the unhealthy lifestyles of workers and poor dietary habits have been recognised as consequences of shift work [51,52]. In studies conducted to date, the authors have observed that night-shift workers are more likely to fail to follow rational nutrition principles than are day-shift workers [53,54]. Moreover, people who perform their professional duties at irregular hours tend to eat more small meals, and they are more likely to snack between meals during ongoing work [55,56]. Furthermore, these workers are more likely to consume larger amounts of high-energy snacks and simple sugars during leisure time than are those working at regular times of the day [56,57,58,59].

In the present study, the observations regarding the intake of sweets, and thus the proportion of added sugars in the diet, are different from those reported by Oliveira L. et al. [16]. The authors of the study reported that the participants’ diets were characterised by an increased proportion of sweets in the diet, due to their responses to stressful situations [16].

As presented in our study, none of the participants in the study exceeded the maximum allowable supply of simple sugars in their diet. However, as we previously wrote, the energy value of the men’s diet is higher; thus, the daily maximum supply of AS and SFA is also higher, relative to a 2000 kcal diet.

In conclusion, the performance of work duties in shifts 24/48 > 10 years by the Wroclaw State Fire Service firefighters is statistically significantly associated with the studied anthropometric characteristics, such as BW, WC, BMI, WHR, SMM, FM, TBW, ECW and ECW ind., and they have higher values compared to men with ST ≤ 10 years. In contrast, diet quality, calculated using the NRF9.3 index, by tertile, was not related to anthropometric measurements or seniority. No participants’ diets exceeded the acceptable maximum AS percentage in the diet, but the diets of only two men in the seniority group > 10 years provided < 1500 mg sodium/day. The men’s diets were additionally low in dietary fibre and calcium. According to the individual-ingredient analysis necessary for calculating NRF9.3, it can be assumed that the men’s dietary patterns are dominated by processed meat (e.g., sausages, pâtés and cold cuts), while vegetables (mainly raw) or dairy products are a small dietary component.

Further studies are needed to analyse the importance of the LS of 24/48 SFS firefighters and/or the number of fire interventions to the minimizing of the risk of metabolic, cancer or cardiovascular disease among officers.

## Figures and Tables

**Table 1:** Recommended Daily Allowance (RDA) and Maximum Daily Value (MDV) developed for study participants’ diets based on guidelines from the European Food and Safety Authority (EFSA) and the Institute of Food and Nutrition (IFN).

DIETARY COMPONENT	RDA	MDV
Protein [g]	10% TM	
Dietary fiber [g]	25	
Ca [mg]	1000	
Mg [mg]	400/420 *	
Fe [mg]	10	
K [mg]	3500	
V. A (µg retinol equivalent)	900	
V. C [mg]	90	
V. E [mg]	10	
Na [mg]		1500
SFA [g]		<10% TM
AS [g]		<10% TM

* the RDA varies according to the age of the study participant; 400 mg/d applies to men < 30 years, while 420 mg/d applies to firefighters ≥ 30 years. TM—total metabolism, Ca—calcium, Mg—magnesium, Fe—iron, K—potassium, Na—sodium, SFA—saturated fatty acids, AS—added sugars.

**Table 2:** Division of the study group into tertiles, relative to the calculated diet quality assessment index NRF9.3.

LOW		MEDIUM		HIGH	
Min.	Max.	Min.	Max.	Min.	Max.
353.65	630.98	631.15	704.40	704.89	846.54

**Table 3:** Comparison of anthropometric parameters of firefighters with shorter and longer length-of-service (≤10 years vs. >10 years).

ANTHROPOMETRIC PARAMETERS	LS ≤ 10 YEARS (*n* = 62)	LS > 10 YEARS (*n* = 71)	*p*
	Mean ± SD/Me (Q1, Q3)	Mean ± SD/Me (Q1, Q3)	
Age [years]	30.08 ± 4.34	40.49 ± 5.08	<0.0001 **
H [cm]	180.60 ± 5.36	179.18 ± 6.77	0.2112 **
BW [kg]	81.95 (76.20, 87.00)	89.00 (80.40, 97.1)	0.0002 *
WC [cm]	89.00 (82.00, 83.00)	96.50 (89.00, 103.00)	<0.0001 *
BMI [kg/m^2^]	25.40 (23.80, 26.80)	28.10 (25.9, 30.00)	<0.0001 *
WHR	0.84 (0.81, 0.86)	0.92 (0.89, 0.96)	<0.0001 *
FM [kg]	17.95 (15.80, 21.40)	22.80 (18.50, 27.20)	<0.0001 *
SMM [%]	43.31 ± 2.42	41.38 ± 3.23	0.1308 *
TBW [l]	45.50 (42.60, 49.20)	43.60 (43.60, 52.10)	0.1308 *
ECW [l]	17.95 (16.90, 19.40)	17.30 (17.30, 21.00)	0.0541 *
ICW [l]	27.35 (25.90, 30.00)	28.40 (26.20, 31.20)	0.2427 *
PBF	21.94 ± 4.06	25.22 ± 5.45	0.0001 **
ECW ind.	0.40 ± 0.01	0.40 ± 0.01	0.0002 **
Imp.[Ω]	410.89 ± 36.03	389.39 ± 43.59	0.0019 **
RA [kg]	50.00 ± 5.92	51.6643 ± 8.75716651	0.2107 **
LA [kg]	53.50 (58.33, 57.67)	57.69 (47.83, 59.20)	0.5333 *

Results are presented as mean values ± standard deviation if the distribution was normal, or as median and quartiles if the distribution was not normal. * Mann Whitney U-test, ** Student’s *t*-test; statistically significant result for *p* < 0.05. H—height, BW—body weight, WC—waist circumference, BMI—body mass index; WHR—waist–hip ratio, SMM—skeletal muscle mass; PBF—percent of body fat, FM—fat mass; TBW—total body water, ECW—extracellular water, ICW—intracellular water, ECW ind.—extracellular water–total body water ratio, RA—average of muscle strength measurements of the right arm, LA—average of muscle strength measurements of the left arm, Imp.—impedance.

**Table 4:** Comparison of dietary energy value, proportion of energy from P, CHO, F, SFA, MUFA, PUFA and macronutrient and micronutrient content of diets of firefighters with short and long service.

DIET COMPONENT	LS ≤ 10 YEARS (*n* = 61)	LS > 10 YEARS (*n* = 69)	*p*
	Mean ± SD/Me (Q1, Q3)	Mean ± SD/Me (Q1, Q3)	
E [kcal]	2754.19 (2280.30, 3360.22)	2851.47 ± 756.4	0.9257 *
P [%]	18.86 ± 3.43	17.45 ± 2.9	0.0119 **
CHO [%]	40.70 (37.58, 44.75)	40.85 (36.82, 45.07)	0.7724 *
F [%]	38.84 (33.63, 42.80)	39.84 (35.05, 45.27)	0.1361 *
SFA [%]	13.77 (10.77, 15.83)	14.52 (12.50, 16.94)	0.1603 *
MUFA [%]	15.38 (13.33, 16.94)	15.90 ± 3.6	0.2444 *
PUFA [%]	5.92 (5.17, 6.92)	6.23 ± 1.4	0.5032 *
P [g]	129.68 (109.53; 162.20)	114.13 (97.37; 154.36)	0.0689 *
CHO [g]	298.55 ± 89.7	267.67 (230.30; 350.13)	0.5756 *
F [g]	117.86 (92.32; 153.42)	128.98 ± 39.7	0.3805 *
SFA [g]	45.60 ± 18.4	48.29 (37.01; 55.51)	0.3413 *
MUFA [g]	47.28 (38.18; 60.81)	51.23 ± 16.9	0.3424 *
PUFA [g]	18.88 (14.97; 22.99)	19.97 ± 7.0	0.6543 *
*n*-6/*n*-3	5.63 (3.93, 7.11)	4.18 (3.41, 6.10)	0.0315 *
Chol. [mg]	665.23 ± 322.4	586.45 ± 261.52	0.1267 **
DF [g]	21.69 (17.40, 29.72)	21.75 (16.45, 26.89)	0.3767 *
V. A [µg]	1293.20 (914.87, 1646.82)	1172.58 (869.36, 1599.56)	0.5077 *
V. B1 [mg]	1.96 ± 0.6	1.81 (1.52, 2.37)	0.9684 *
V. B2 [mg]	2.76 ± 0.8	2.36 (1.95, 3.25)	0.1821 *
V. B3 [mg]	32.66 (23.67, 38.79)	27.84 (21.98, 36.56)	0.1090 *
V. B5 [mg]	0.82 (0.47, 1.35)	0.92 (0.52, 1.49)	0.3653 *
V. B6 [mg]	3.04 (2.46, 3.49)	2.59 (2.06, 3.50)	0.0434 *
V. B12 [µg]	6.36 (4.62, 8.24)	5.94 (4.11, 8.28)	0.4540 *
V. C [mg]	144.76 (94.36, 196.62)	107.35 (71.86, 149.14)	0.0253 *
V. D [µg]	4.62 (3.11, 6.38)	5.08 (3.82, 8.24)	0.1528 *
V. E [mg]	15.76 (11.81, 20.59)	15.76 (12.61, 20.73)	0.7458 *
DFE [µg]	442.20 (358.06, 574.67)	363.82 (290.89, 475.90)	0.0169 *
Ca [mg]	1007.38 ± 341.4	864.54 (691.04, 1150.71)	0.2570 *
Fe [mg]	16.35 ± 4.6	14.64 (12.38, 17.80)	0.1987 *
Mg [mg]	411.72 (341.49, 529.78)	372.38 (311.45, 511.57)	0.2416 *
K [mg]	4230.20 ± 1044.9	3663.00 (3172.11, 5033.97)	0.2287 *
Na [mg]	3461.78 ± 1037.2	3241.24 (2556.04, 4035.50)	0.4359 *
Zn [mg]	14.81 (12.84, 18.24)	13.81 (12.19, 16.77)	0.1508 *
Caf. [mg]	108.10 (34.26, 160.14)	128.11 (60.05, 200.38)	0.1031 *
NRF9.3	662.50 ± 103.1	664.78 (602.67, 710.41)	0.3805 *

Results are presented as mean values ± standard deviation if the distribution was normal or as median and quartiles if the distribution was not normal. * Mann–Whitney U-test, ** Student’s *t*-test; statistical significance, statistically significant result for *p* < 0.05. E—energy value, P—protein, CHO—carbohydrates, F—fat, SFA—saturated fatty acids, MUFA—monounsaturated fatty acids, PUFA—polyunsaturated fatty acids, *n*-6—polyunsaturated fatty acids of the omega-6 family, *n*-3—polyunsaturated fatty acids of the omega-3 family, DF—dietary fibre, Fol.—folate, Chol.—cholesterol, Caf.—caffeine, DFE—dietary folate equivalent.

**Table 5:** Comparison of the individual components of the diet quality indicator NRF9.3 relative to length-of-service.

TM AND NRF9.3 INDEX COMPONENTS	LS ≤ 10 YEARS (*n* = 61)	LS > 10 YEARS (*n* = 69)	*p*
	Mean ± SD/Me (Q1, Q3)	Mean ± SD/Me (Q1, Q3)	
TM [kcal]	2873.50 (2784.25, 3018.75)	2857.93 ± 282.1	0.1090
DF [% RDA]	86.77 (69.59, 100.00)	87.01 (65.79, 100.00)	0.3425
P [% RDA]	100.00 (100.00, 100.00)	100.00 (100.00, 100.00)	0.9477
Ca [% RDA]	93.90 (76.64, 100.00)	86.45 (69.10, 100.00)	0.2623
V. C [% RDA]	100.00 (100.00, 100.00)	100.00 (85.78, 100.00)	0.2358
Mg [% RDA]	100.00 (85.37, 100.00)	93.09 (77.86, 100.00)	0.0979
Fe [% RDA]	100.00 (100.00, 100.00)	100.00 (100.00, 100.00)	0.4568
K [% RDA]	100.00 (96.88, 100.00)	100.00 (90.63, 100.00)	0.2285
V. E [% RDA]	100.00 (100.00, 100.00)	100.00 (100.00, 100.00)	0.6095
V. A [% RDA]	100.00 (100.00, 100.00)	100.00 (97.62, 100.00)	0.7930
SFA [% RDA]	133.12 (94.28, 173.32)	149.93 ± 50.5	0.1791
SFA to NRF [% RDA]	33.12 (0.00, 73.32)	47.07 (16.21, 77.32)	0.1987
AS [% RDA]	5.17 (1.39, 13.68)	2.39 (0.46, 10.40)	0.0777
AS DO NRF [% RDA]	0.00 (0.00, 0.00)	0.00 (0.00, 0.00)	-
Na [% RDA]	230.79 ± 69.1	216.14 (173.90, 269.03)	0.4870
Na to NRF [% RDA]	130.79 ± 69.1	116.14 (73.90, 169.03)	0.4870
NRF9	859.31 (808.82, 896.24)	844.56 (800.82, 878.77)	0.1168
NRF3	160.63 (98.95, 243.08)	168.10 (108.38, 226.23)	0.8887
NRF9.3	662.50 ± 103.1	664.78 (602.67, 710.41)	0.3805

Results are presented as mean values ± standard deviation if the distribution was normal or as median and quartiles if the distribution was not normal. p—statistical significance, statistically significant result for *p* < 0.05. TM—total metabolism; P—protein, DF—dietary fibre, SFA—saturated fatty acids, AS—added sugars, Ca—calcium, Mg—magnesium, Fe—iron, K—potassium, Na—sodium; NA to NRF—excess sodium in the diet, relative to the RDA, taken to calculate the NRF9.3 index; SFA to NRF—excess of saturated fatty acids in relation to RDA, used to calculate the NRF9.3 index; AS to NRF—excess of added sugars relative to the RDA, taken to calculate the NRF9.3 index; NRF9—subscore based on 9 nutrients to be encouraged; NRF3—subscore based on 3 nutrients to be limited, NRF9.3—Nutrient Rich Food Index 9.3.

**Table 6:** Comparison of the percentages of participants from the LS ≤ 10 years and LS > 10 years groups whose diets provided the requirements for each of the required dietary components and observed the dietary component restriction according to dietary recommendations.

NRF9.3 INDEX COMPONENTS	LS ≤ 10 YEARS (*n* = 61)		LS > 10 YEARS (*n* = 69)		χ^2^	*p*
	%n	y	%n	y		
DF	44.3	27	29.0	20	3.27	0.0704
P	98.4	60	98.6	68	0.01	0.9300
Ca	44.3	27	39.1	27	0.35	0.5535
V. C	75.4	46	65.2	45	1.60	0.2057
Mg	55.7	34	42.0	29	2.44	0.1186
Fe	93.4	57	89.9	62	0.54	0.4633
K	68.9	42	59.4	41	1.25	0.2640
V. E	88.5	54	91.3	63	0.28	0.5980
V. A	75.4	46	73.9	51	0.04	0.8449
SFA	31.1	19	14.5	10	5.18	0.0228
AS	100.0	61	100.0	69	-	-
Na	0.0	0	2.9	2	-	0.4980

n—the number of people in the group; y—the number of diets of study participants fulfilling the requirement for the dietary component in the study; χ^2^—χ^2^ test; p—statistical significance, statistically significant result for *p* < 0.05; DF—dietary fiber; P—protein; SFA—saturated fatty acids; AS—added sugars; Ca—calcium; Mg—magnesium; Fe—iron; K—potassium; Na—sodium.

**Table 7:** Effect of length-of-service (LS ≤ 10 years, LS > 10 years) and diet quality (NRF9.3-C: Low, Medium, High) on anthropometric characteristics (SMM, PBF, TBW, Imp., LA, LS) by ANOVA.

DEPENDENT VARIABLE PREDICTOR	PREDICTOR VALUE	B	95% CONFIDENCE INTERVAL (B)	BETA (ß)	95% CONFIDENCE INTERVAL (ß)	t	*p*	P	R^2^
SMM [%]									
LS	1	−2.18	(−3.94; −0.42)	−0.36	(−0.65; −0.07)	−2.45	0.016	0.006	0.09
NRF9.3-C	Low	−0.72	(−2.48; 1.04)	−0.11	(−0.39; 0.16)	−0.81	0.418		
NRF9.3-C	Medium	−0.37	(−2.15; 1.42)	−0.06	(−0.34; 0.22)	−0.41	0.685		
LS:NRF9.3-C	1:Low	0.23	(−2.22; 2.69)	0.03	(−0.29; 0.35)	0.19	0.853		
LS:NRF9.3-C	1:Medium	0.24	(−2.24; 2.73)	0.03	(−0.29; 0.35)	0.19	0.847		
PBF [%]									
LS	1	3.69	(0.73; 6.65)	0.36	(0.07; 0.66)	2.46	0.015	0.005	0.09
NRF9.3-C	Low	1.18	(−1.78; 4.15)	0.11	(−0.17; 0.39)	0.79	0.430		
NRF9.3-C	Medium	0.63	(−2.37; 3.63)	0.06	(−0.22; 0.34)	0.42	0.678		
LS:NRF9.3-C	1:Low	−0.35	(−4.48; 3.78)	−0.03	(−0.35; 0.29)	−0.17	0.869		
LS:NRF9.3-C	1:Medium	−0.42	(−4.60; 3.75)	−0.03	(−0.35; 0.29)	−0.20	0.841		
TBW [%]									
LS	1	−2.64	(−4.78; −0.5)	−0.36	(−0.65; −0.07)	−2.44	0.016	0.005	0.09
NRF9.3-C	Low	−0.86	(−3.00; 1.28)	−0.11	(−0.39; 0.17)	−0.80	0.426		
NRF9.3-C	Medium	−0.44	(−2.61; 1.73)	−0.06	(−0.34; 0.22)	−0.40	0.687		
LS:NRF9.3-C	1:Low	0.25	(−2.73; 3.23)	0.03	(−0.29; 0.35)	0.17	0.866		
LS:NRF9.3-C	1:Medium	0.28	(−2.74; 3.29)	0.03	(−0.29; 0.35)	0.18	0.855		
Imp. [Ω]									
LS	1	−1.81	(−25.62; 22)	−0.02	(−0.31; 0.27)	−0.15	0.881	0.002	0.10
NRF9.3-C	Low	35.89	(12.08; 59.7)	0.42	(0.14; 0.69)	2.98	0.003		
NRF9.3-C	Medium	17.20	(−6.94; 41.34)	0.20	(−0.08; 0.47)	1.41	0.161		
LS:NRF9.3-C	1:Low	−36.67	(−69.86; −3.48)	−0.35	(−0.67; −0.03)	−2.19	0.031		
LS:NRF9.3-C	1:Medium	−25.75	(−59.33; 7.82)	−0.24	(−0.56; 0.07)	−1.52	0.131		
LA [kg]									
LS	1	2.71	(−2.17; 7.58)	0.17	(−0.14; 0.48)	1.10	0.274	0.353	0.00
NRF9.3-C	Low	−0.53	(−5.40; 4.35)	−0.03	(−0.33; 0.26)	−0.21	0.831		
NRF9.3-C	Medium	−0.69	(−5.63; 4.25)	−0.04	(−0.34; 0.25)	−0.28	0.781		
LS:NRF9.3-C	1:Low	−1.25	(−8.01; 5.51)	−0.06	(−0.40; 0.28)	−0.37	0.716		
LS:NRF9.3-C	1:Medium	−4.70	(−11.56; 2.17)	−0.23	(−0.56; 0.11)	−1.35	0.178		
SBP [mmHg]									
LS	1	4.75	(−4.13; 13.64)	0.17	(−0.14; 0.47)	1.06	0.291	0.288	0.01
NRF9.3-C	Low	1.18	(−7.58; 9.94)	0.04	(−0.25; 0.33)	0.27	0.791		
NRF9.3-C	Medium	−3.14	(−12.02; 5.74)	−0.10	(−0.39; 0.19)	−0.70	0.485		
LS:NRF9.3-C	1:Low	−1.82	(−14.12; 10.48)	−0.05	(−0.39; 0.29)	−0.29	0.770		
LS:NRF9.3-C	1:Medium	4.79	(−7.71; 17.29)	0.13	(−0.21; 0.46)	0.76	0.450		
PULSE [ud/min]									
LS	1	0.46	(−7.28; 8.19)	0.02	(−0.3; 0.34)	0.12	0.907	0.876	−0.03
NRF9.3-C	Low	3.67	(−3.96; 11.30)	0.14	(−0.16; 0.45)	0.95	0.343		
NRF9.3-C	Medium	2.09	(−5.65; 9.82)	0.08	(−0.22; 0.38)	0.53	0.594		
LS:NRF9.3-C	1:Low	−2.46	(−13.11; 8.20)	−0.08	(−0.43; 0.27)	−0.46	0.649		
LS:NRF9.3-C	1:Medium	1.38	(−9.44; 12.21)	0.04	(−0.3; 0.39)	0.25	0.801		

B—predictor coefficient, β—standardized predictor coefficients, t—test value, *p*—statistical significance, P—model statistical significance, R^2^—determination coefficient, statistically significant result for *p* < 0.05. SMM—skeletal muscle mass, PBF—percent of body fat, TBW—total body water, Imp.—impedance, LA—average of muscle strength measurements of the left arm, SBP—systolic blood pressure. Reference levels: LS—“0”, NRF9.3-C—“High”, “LS: NRF9.3-C” indicates the interaction between the predictors LS and NRF9.3-C.

**Table 8:** Comparison of the association of length-of-service (LS ≤ 10 vs. LS > 10 years) and diet quality (NRF9.3-C: Low, Medium, High) on anthropometric parameters according to a ‘robust ANOVA’ (for details, see ‘Statistical Analysis’).

ANTHROPOMETRIC PARAMETERS	LS		NRF9.3-C		LS:NRF9.3-C	
	Factor	*p*	Factor	*p*	Factor	*p*
BW [kg]	15.329	0.001	1.090	0.589	1.864	0.407
WC [cm]	20.621	0.001	3.251	0.214	0.311	0.859
BMI [kg/m^2^]	25.055	0.001	0.228	0.895	1.3660	0.517
WHR	84.228	0.001	2.022	0.377	1.464	0.942
SMM [kg]	4.207	0.045	1.005	0.603	2.203	0.349
RA [kg]	0.360	0.551	3.784	0.167	0.165	0.923
FM [kg]	18.535	0.001	1.494	0.486	0.403	0.822
TBW [l]	4.856	0.032	1.078	0.593	2.134	0.360
ECW [l]	4.158	0.017	0.868	0.657	2.169	0.356
ICW [l]	3.196	0.079	1.294	0.534	2.477	0.306
ECW ind.	18.065	0.001	0.406	0.820	1.400	0.508
DBP [mmHg]	3.457	0.068	2.664	0.280	0.525	0.774

BW—body weight, WC—waist circumference, BMI—body mass index; WHR—waist–hip ratio, SMM—skeletal muscle mass; PBF—percent of body fat, FM—fat mass; TBW—total body water, ECW—extracellular water, ICW—intracellular water, ECW ind.—extracellular water–total body water ratio, RP—average of muscle strength measurements of the right arm, DBP—diastolic blood pressure; statistically significant result for *p* < 0.05.

## Data Availability

The data presented in this study are available on request from the corresponding author. The data are not publicly available due to participants’ sensitive data.

## Supplementary Materials

The following supporting information can be downloaded at: https://www.mdpi.com/article/10.3390/nu15184029/s1, Table S1: Calculation method of the NRF9.3 index based on data obtained from one of the study participants, aged 28 years.
