# Supplementary material for: Association of the Length of Service of 24/48 Firefighters with the Quality of Their Diet and Selected Anthropometric Parameters
Source: Nutrients. 2023 Sep 17;15(18):4029. doi: 10.3390/nu15184029 (PMC10536748; doi:10.3390/nu15184029)
Supplement: Supplementary file 1 [file nutrients-15-04029-s001.zip › nutrients-2597828-supplementary.pdf]

The detailed calculation method of the index, is shown in Table S1, using data obtained from one of the study participants as an example.

Table S1 Calculation method of the NRF9.3 index based on data obtained from one of the study participants, aged 28 years.

**TM = BMR × PAL = 1654 kcal × 1,75 = 2894,5 kcal**					
**DIETARY COMPONENT**	**RDA**	**DI**	**%RDA**	**CM**	**VFC**
Protein [g]	72,36	94,12	130,07	%RDA > 100; the percentage of total fulfilment of the daily requirement for this component has been assumed	100,00
Dietary fibre [g]	25,00	28,01	112,04		100,00
Ca [mg]	1000	1109,42	110,94		100,00
V. C [mg]	90,00	119,42	132,69		100,00
Mg [mg]	400,00	452,68	113,17		100,00
Fe [mg]	10,00	12,85	128,53		100,00
K [mg]	3500,00	3669,53	104,84		100,00
V. E [mg]	10,00	21,26	212,57		100,00
V. A [µg retinol equivalent]	900,00	836,21	92,91		92,91
Na [mg]	1500,00	2241,69	149,45	149,45-100= 49,45	49,45
SFA [g]	32,16	43,30	134,63	134,63-100=34,63	34,63
AS [g]	74,43	31,85,	42,79	42,79-100 = -57,21 <0 – assumed value 0	0
NRF9	100,00+100,00+100,00+100,00+100,00+100,00+100,00+100,00+92,91= 892,91				
NRF3	49,45+34,63+0= 84,08				
NRF9.3	892,91-84,08=808,83				

BMR – Basal Metabolic Rate, TM – total metabolism, PAL – Physical Activity Level, DI – dietary intake, %RDA – percentage of Recommended Daily Allowance, CM – calculation method; VFC – value to be used in the calculation of NRF9.3, Ca – calcium, Mg – magnesium, Fe – iron, K – potassium, Na – sodium, SFA – saturated fatty acids, AS - added sugars NRF9 – subscore based on 9 nutrients to encourage; NRF3 – subscore based on 3 nutrients to limit;
